# Perceived barriers and facilitators to medical students participating in volunteer services for older adults with disabilities: a qualitative descriptive study

**DOI:** 10.3389/fpubh.2026.1918396

**Published:** 2026-10-13

**Authors:** Tong Liu, Runli Yang, Hui Dong, Ling Zhong, Xinle Zhong, Hengying Chen, Jun Wang, Qinghua Zhao, Yuan Jia, Mingzhao Xiao

**Affiliations:** 1 School of Nursing, Chongqing Medical University, Chongqing, China; 2 Nursing Research Center, The First Affiliated Hospital of Chongqing Medical University, Chongqing, China; 3 Department of Nursing, The First Affiliated Hospital of Chongqing Medical University, Chongqing, China; 4 Department of Academic Affairs, Chongqing Nursing Vocational College, Chongqing, China

**Keywords:** barriers and facilitators, COM-B model, medical students, older adults with disabilities, volunteer services

## Abstract

**Background:**

With global aging accelerating, volunteer services have become a cornerstone for sustaining care for older adults with disabilities (older adults aged 60 and above with age-related functional limitations). Medical students play a unique role in this system due to their professional competencies. However, they encounter particular environmental and psychological pressures that impact their sustained engagement. Existing literature has mostly focused on general motivations, while the barriers and facilitators influencing sustained participation have not been sufficiently explored through the application of a theoretical framework. This study aims to explore the facilitators and barriers of medical students participating in volunteer services for older adults with disabilities.

**Methods:**

A qualitative descriptive study was conducted using purposive sampling from two medical schools in Southwest China. Sixteen semi-structured interviews were conducted between October 2025 and January 2026 based on the Capability, Opportunity, and Motivation-Behavior (COM-B) model. Data analysis utilized the framework approach, with the Theoretical Domains Framework (TDF) used to map behavioral factors.

**Results:**

Analysis revealed nine core themes regarding medical students’ perceptions of the barriers and facilitators to volunteering with older adults with disabilities within the COM-B model and the TDF framework. Facilitators included strong professional identity and empathy, social influence and interpersonal support, and incentive structures with career ambitions. Barriers were identified as the gap between theoretical knowledge and practice, physical stamina, and emergency decision-making pressures, information accessibility issues, resource limitations, and emotional experiences, coupled with psychological obstacles. A dilemma of “high intention, low efficacy” was observed.

**Conclusion:**

The present findings highlight the complex relationship between students’ professional aspirations and practical constraints and could contribute to the development of more effective volunteer service models for older adults with disabilities. Medical educational institutions and community organizations should implement simulation-based training to bridge the theory-practice gap, establish psychological support systems to enhance resilience, and optimize resource allocation. These measures are necessary to transform medical students’ motivation into sustainable practical capability.

## 1 Introduction

With the development of the global economy, the aging population has become a significant global public health challenge, and the increasing disability rate among older adults has become an urgent social issue (1). The World Health Organization’s Global Report on Health Equity for Persons with Disabilities (2022) indicates that around 1.3 billion individuals, or 1 in 6 of the global population, experience substantial disability, with older adults being disproportionately impacted due to chronic conditions and functional decline (2). In China, the situation is particularly critical due to the rapid pace of demographic changes. The Fourth National Sample Survey on the Living Conditions of the older adults in Urban and Rural Areas projected that the population of older adults with disabilities in China would reach 61.68 million by 2030 (3). In this study, the term “older adults with disabilities” refers to community-dwelling older adults aged 60 years and older who experience limitations in activities of daily living (ADLs) due to chronic conditions or age-related functional decline (3). This demographic trend imposes a significant strain on the official healthcare system and family caregivers (4).

Research indicates that formal care resources are often insufficient to meet the surging demand, making volunteerism a cornerstone for the sustainability of long-term care (5). Medical students, including those in clinical medicine, nursing, and rehabilitation disciplines, hold a distinctive and strategic role among the various volunteer groups. In China, medical education is increasingly emphasizing the integration of professional skills with medical humanism and social responsibility (6). As compared to general volunteers, medical students possess essential medical knowledge and basic caregiving skills, enabling them to provide more professional and safe assistance to older adults with disabilities. Participating in such services fulfills a twofold function. For older adults, students can offer high-quality companionship and health guidance that lay volunteers may lack. For the students, early exposure to older adults with disabilities helps bridge classroom-based explicit theoretical knowledge, such as geriatric disease concepts and formal care protocols, with practical application. It also facilitates the acquisition of practical and tacit knowledge, the “tacit dimension” described by Polanyi (7), through experiences such as interpreting non-verbal communication, exercising clinical judgment in unpredictable caregiving situations, and developing empathy toward service recipients. At the same time, such exposure reduces potential ageist stereotypes and fosters a strong sense of professional identity and empathy.

Providing volunteer services to older adults with disabilities presents a range of unique challenges compared to general community service, as it takes place in a complex physiological and psychological environment. Students often encounter high-pressure scenarios, such as communicating with older adults with cognitive impairments (dementia) or assisting them with physically demanding tasks like transfers and toileting (8). These pressures can lead to anxiety and feelings of helplessness, obstructing continuous engagement. Most previous research has focused on quantitative surveys of general altruistic motivation or participation rates, with less attention paid to the specific barriers and facilitating factors associated with helping older adults with disabilities (9, 10).

Furthermore, the underlying mechanisms by which students choose to persist or withdraw have not been thoroughly explored from a systematic theoretical perspective, making it difficult to design effective educational interventions. Therefore, this study uses the Competency, Opportunity, and Motivation Model (COM-B) with the Theoretical Domain Framework (TDF) to explore the barriers and facilitators perceived by medical students when participating in volunteer services for older adults with disabilities, aiming to enhance volunteer service initiatives, promote student development, and improve the quality of care for older adults with disabilities.

## 2 Methods

### 2.1 Design

A qualitative descriptive study involving medical students was conducted using semi-structured interviews. Unlike phenomenology, which interprets the underlying philosophical meaning of lived experiences, or a case study, which examines a specific bounded system, qualitative description is optimal for obtaining straightforward and comprehensive accounts of concrete phenomena (11); this design aligns perfectly with our objective. Furthermore, the primary advantage of utilizing semi-structured interviews lies in allowing researchers to explore pre-defined topics derived from the COM-B framework while retaining the flexibility to follow up on participants’ accounts and investigate unanticipated barriers and facilitators in depth (12). Ultimately, this comprehensive qualitative approach is well-suited for examining the factors influencing students’ sustained participation in volunteer services for older adults with disabilities and can directly inform the design of future interventions (13).

### 2.2 Conceptual framework

To provide both theoretical and pragmatic guidance for this study, we used two complementary frameworks: the Capability, Opportunity, and Motivation Model of Behavior (COM-B) (14) and the Theoretical Domains Framework (TDF) (15) (Figure 1). Situated at the center of the Behavior Change Wheel, the COM-B model asserts that behavior is influenced by the interplay of Capability, Opportunity, and Motivation, with Capability and Opportunity playing crucial roles as mediators of behavior change. The TDF refines the granularity of this behavioral analysis by detailing the COM-B components across fourteen specific domains, such as beliefs about capabilities and environmental context, which correspond directly to the COM-B categories, thereby delineating the unique cognitive and environmental determinants of behavior (16, 17). Although these frameworks have been extensively applied in various healthcare settings, there is a scarcity of research applying this combined approach to investigate student volunteering specifically for older adults with disabilities. Consequently, this study utilizes them to identify specific barriers and facilitators to define precisely what needs to shift to achieve sustained engagement and improve care quality for this vulnerable population.

**Figure 1:**
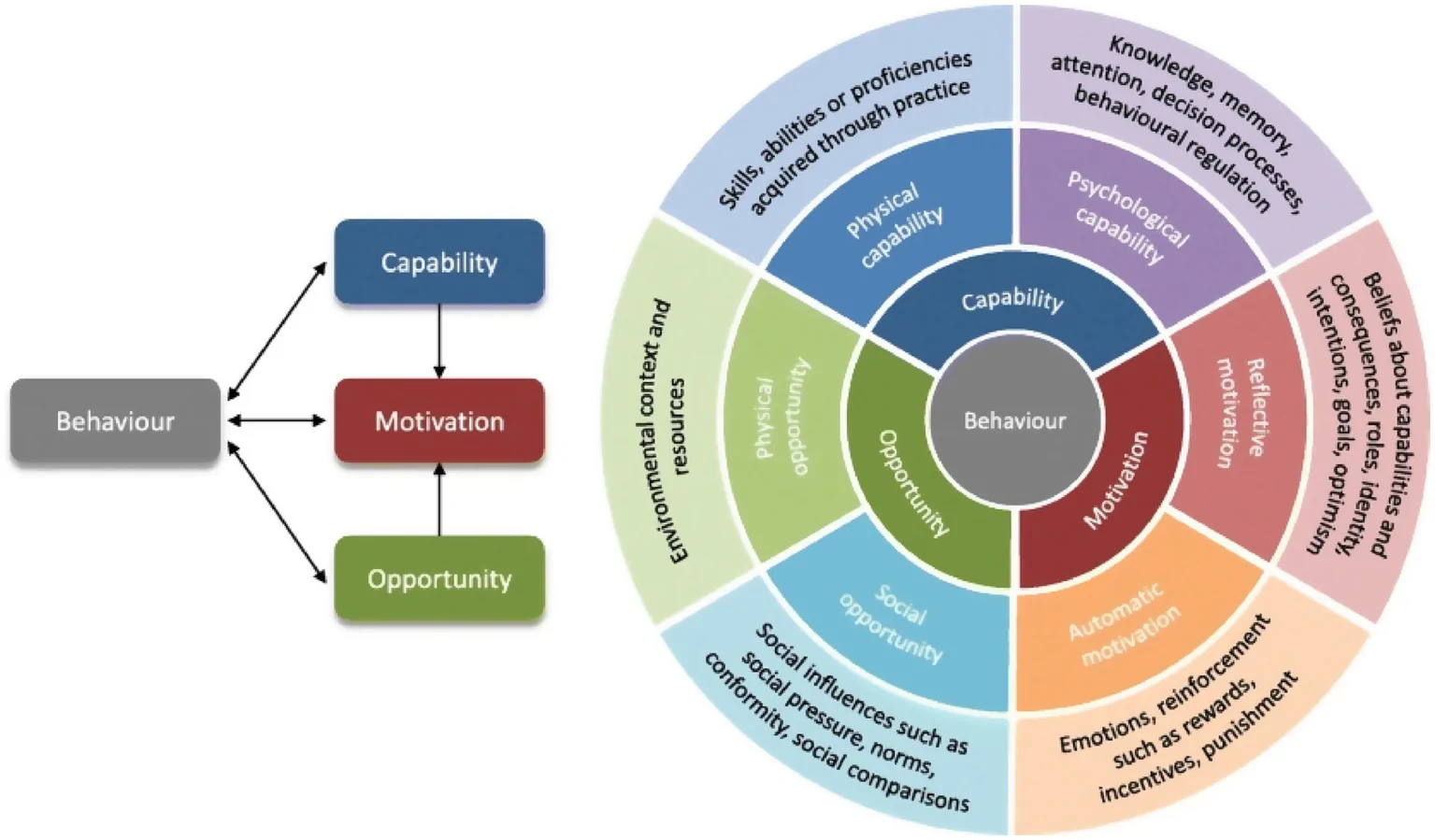
COM-B domains and TDF domains. Reproduced with permission from McDonagh et al. (39).

### 2.3 Study setting and participant

This study was conducted at Chongqing Medical University and Chongqing Nursing Vocational College in Chongqing, between October 2025 and January 2026. Purposive sampling was employed to recruit participants from the majors of clinical medicine, nursing, rehabilitation, social work (medical social work), and geriatric health management among other health-related majors. These participants were selected as students because they represent the future workforce most likely to engage in careers related to geriatric care. Maximum variation sampling was employed to encompass a wide range of opinions, ensuring diversity in terms of age, gender, and major. We recruited potential participants through various methods, including posting posters on campus bulletin boards, receiving recommendations from volunteer team teachers, and spreading recruitment information through student WeChat groups.

The inclusion criteria were as follows: (1) aged over 18 years; (2) participation in volunteering services for older adults with disabilities at least three times (volunteer services referred to community and home-based activities, including home visits and companionship, chronic disease health education, guided rehabilitation exercises, basic care demonstrations such as pressure ulcer prevention, caregiving skills training for family caregivers, and emotional support); and (3) willingness to share volunteering experiences with researchers. Exclusion criteria were as follows: (1) had suspended their studies or were on leave; (2) participated in volunteering sessions lasting less than one hour on average; or (3) had a diagnosed cognitive impairment or psychiatric condition that prevented them from communicating clearly during the interview.

Recruitment was terminated once data sufficiency was achieved, with sample adequacy evaluated according to the five essential components of information power (18): (1) study aim, (2) sample specificity, (3) use of established theory, (4) quality of dialogue, and (5) analysis strategy. The ultimate sample size was evaluated based on the principle of information power. The cohort was considered suitable for exploratory analysis due to its narrow study scope, the high specificity of the interviews, the strong theoretical foundation from our previous quantitative research, and the quality of the discourse collected.

### 2.4 Data collection

The semi-structured interview guide (Supplementary file 1) was developed based on the research objectives and informed by the COM-B model. The guide was structured into four primary sections: demographic characteristics and the three domains of the behavioral model: Capability, Opportunity, and Motivation. The inquiry focused on participants’ professional knowledge and physical stamina (Capability), external support systems and time management (Opportunity), and emotional responses or sense of responsibility regarding volunteering for older adults with disabilities (Motivation). Before the primary interviews, two pilot interviews were conducted with students excluded from the final sample to test the clarity and flow of the questions. Minor adjustments were made to the guide based on their feedback.

A total of 16 in-depth interviews were conducted by the first author between October and January 2026. A team of three researchers, all trained in qualitative interviewing techniques, was present during each session. The first author led the primary interview and questioning, while a second researcher managed the audio recording and documented non-verbal cues. The third researcher was responsible for assisting two other researchers. All sessions were audio-recorded to ensure accuracy. The interviews were conducted in quiet and private classrooms within the campus to ensure anonymity and promote open discourse. Before the interview began, potential participants received a comprehensive information sheet and were allowed to raise queries. Thereafter, the researcher dedicated around 5–10 min to establishing rapport through casual conversation. This preliminary interaction was designed to alleviate potential nervousness and build a trusting relationship. Written informed consent was obtained immediately before data collection began. Each interview lasted between 30 and 60 min and was considered concluded when all key dimensions of the COM-B framework had been thoroughly explored, and the participant had no further insights to provide. Demographic information, including grade, major, and previous volunteering experience, was recorded at the start of the session.

The sample size was initially estimated at 10–15 interviews based on recommendations by Francis et al. (19) and previous health research using the TDF and COM-B models (20–22). However, the final determination was governed by the principle of data sufficiency rather than traditional data saturation. We prioritized sufficiency to ensure the dataset had sufficient depth and variety to effectively address the study issues without the strict necessity of proving theoretical redundancy. By the 16th interview, the research team, having conducted data analysis concurrently with recruitment, concluded that the gathered data were sufficiently substantial. The dataset was deemed robust enough to not only identify key barriers and facilitators but also to capture the complex opinions of the participants, satisfying the study’s exploratory goals.

### 2.5 Data analysis

All interviews were audio-recorded and subsequently transcribed verbatim into Mandarin Chinese. To ensure the accuracy of the textual data, the first author reviewed all transcripts against the original audio recordings. Data analysis was conducted using the Framework Method (23), which allowed for a systematic exploration of the data. This method facilitated a combined analytical strategy where themes were identified deductively based on the pre-existing domains of the COM-B model and inductively from the participants’ raw experiences.

The analytical process began with familiarization with the data. The first author and a senior researcher with experience in qualitative data analysis together immersed themselves in the data by repeatedly reviewing the transcripts and recordings to gain a comprehensive understanding. To ensure coding reliability, two researchers independently analyzed the initial three transcripts. Subsequently, they evaluated their coding decisions to ensure consistency. Based on this initial comparison, a preliminary coding framework was developed. This framework incorporated both the theoretical constructs of Capability, Opportunity, and Motivation and new concepts related to student volunteering experiences.

NVivo 12 software was utilized to manage the data and apply the coding framework to the remaining transcripts. This process was iterative and flexible, allowing for the refinement of codes as new patterns emerged. Researchers categorized associated codes to establish themes and sub-themes. These were then mapped onto the specific components of the COM-B model. When the correlation of codes to certain domains was unclear, the research team held consensus meetings to discuss and resolve the discrepancies. Finally, the data were summarized in a framework matrix. This matrix facilitated the comparison of cases and the selection of representative quotes to illustrate the findings regarding the barriers and facilitators to volunteering for older adults with disabilities.

### 2.6 Ethical considerations

This study was performed in accordance with the principles of the WMA Declaration of Helsinki (24). Ethical approval for the research protocol was granted by the First Affiliated Hospital of Chongqing Medical University Ethics Committee on 22 September 2025 (No. 2025-716-01). Informed consent was obtained from all individual participants included in the study. This study was reported according to the Consolidated Criteria for Reporting Qualitative Research (COREQ) reporting guideline (25).

Several ethical issues were addressed during the study. First, although recruitment involved recommendations from volunteer team teachers, all participants were explicitly informed that they had no direct teaching or assessment relationship with the researchers and that their decision to participate or withdraw would not affect their academic standing or course grades, thereby minimizing perceived coercion. Second, as the interviews covered emotionally sensitive topics such as incontinence care and fear of death, the interviewer paused whenever participants showed signs of emotional distress and allowed them to skip questions or terminate the interview; information about psychological counseling services was also provided when necessary. Third, participants were reminded not to disclose any identifying information about the older adults they served, and the team removed all such details during transcription. All research materials were kept strictly confidential and securely stored on password-protected, encrypted institutional servers accessible only to core research team members.

### 2.7 Trustworthiness and reflexivity

To ensure data validity and establish the overall trustworthiness of the findings, Lincoln and Guba’s criteria of credibility, dependability, confirmability, and transferability were used to assess the rigor of the study (26). To establish credibility, member checking was utilized, allowing participants to examine their interview transcripts and the emerging themes. Regular peer debriefing sessions were held among the research team to discuss emerging themes and contest initial interpretations, thereby reducing individual researcher bias. Dependability was established through meticulous documentation of the complete research process and analytical progression. This documentation recorded the entire research process, including raw data, coding notes, and decisions regarding the refinement of the thematic framework. Confirmability was achieved by an independent coding method and the triangulation of data analysis conducted by several researchers. This ensured that the conclusions were based exclusively on the participants’ accounts, rather than the researchers’ biases. Transferability was addressed by providing a thick description of the research setting at Chongqing Medical University and Chongqing Nursing Vocational College, as well as the specific characteristics of the participants. This enables other researchers to determine the applicability of the findings to similar circumstances.

Regarding reflexivity, the research team remained conscious of their backgrounds and possible biases during the study. Significantly, all researchers involved in the interviews indicated that they had no direct teacher-student contact with the participants. The first author, who conducted the interviews, engaged in continuous self-reflection and maintained a reflexive diary to set aside pre-existing beliefs regarding student volunteering and senior care. This reflexive approach ensured that the interview questions regarding capability, opportunity, and motivation were explored openly without leading the participants. Consequently, the analysis remained grounded in the genuine viewpoints of the students.

## 3 Results

### 3.1 Participant characteristics

Data sufficiency was achieved after 16 interviews. Each interview lasted 30–60 min, with an average of approximately 45 min. Participants (*N* = 16) ranged in age from 18 to 22 (M = 19.7 years). A total of 10 participants were female, and 6 were male. Participants came from various academic disciplines and specialties within the medical and health fields, including Nursing (*n* = 5), Clinical Medicine (*n* = 2), Social Work (*n* = 2), and Rehabilitation (Community Rehabilitation and TCM Rehabilitation Technology, *n* = 2). The remaining participants represented diverse backgrounds such as Public Health (*n* = 1), Pharmacy (*n* = 1), Geriatric Health and Management (*n* = 1), Midwifery (*n* = 1), and Traditional Chinese Medicine (*n* = 1). Regarding volunteering experience, service duration ranged from 0.5 to 4 years (*M* = 1.9 years), and most participants (*n* = 13) reported serving 2 h per session. Table 1 shows the detailed demographic characteristics of the participants, and Supplementary Table S1.

**Table 1:** Participants’ demographic characteristics (*N* = 16).

Participant	Gender	Age	Major	Years of volunteer service (years)	Hours of service per instance (hours)
P1	F	22	Nursing	4	2
P2	M	18	Clinical Medicine	0.5	2
P3	F	21	Nursing	4	2
P4	F	21	Nursing	2	2
P5	F	20	Social Work (Medical Social Work)	2	2
P6	M	20	Clinical Medicine	2	2
P7	F	20	Nursing	2	2
P8	F	19	Traditional Chinese Medicine Rehabilitation Techniques	3	2
P9	M	19	Pharmacy	1	3
P10	M	18	Geriatric Health Care and Management	0.5	2
P11	F	19	Community Rehabilitation	1	2
P12	M	22	Public Health	2	2
P13	F	20	Social Work (Medical Social Work)	1	2
P14	F	19	Nursing	3	2
P15	F	19	Midwifery	2	3
P16	M	18	Traditional Chinese Medicine	1	3

### 3.2 Research findings

Based on the COM-B model and the Theoretical Domains Framework (TDF), the study revealed participants’ opinions on the barriers and facilitators to sustained participation in volunteer services for older adults with disabilities. The word cloud in Figure 2 visually presents the high-frequency concepts and core focal points extracted from the qualitative data. Furthermore, the findings are categorized into three dimensions: Capability, Opportunity, and Motivation, each containing unique sub-themes. A detailed summary of the identified themes, mapped to the corresponding COM-B and TDF domains, is presented in Figure 3 and Table 2.

**Figure 2:**
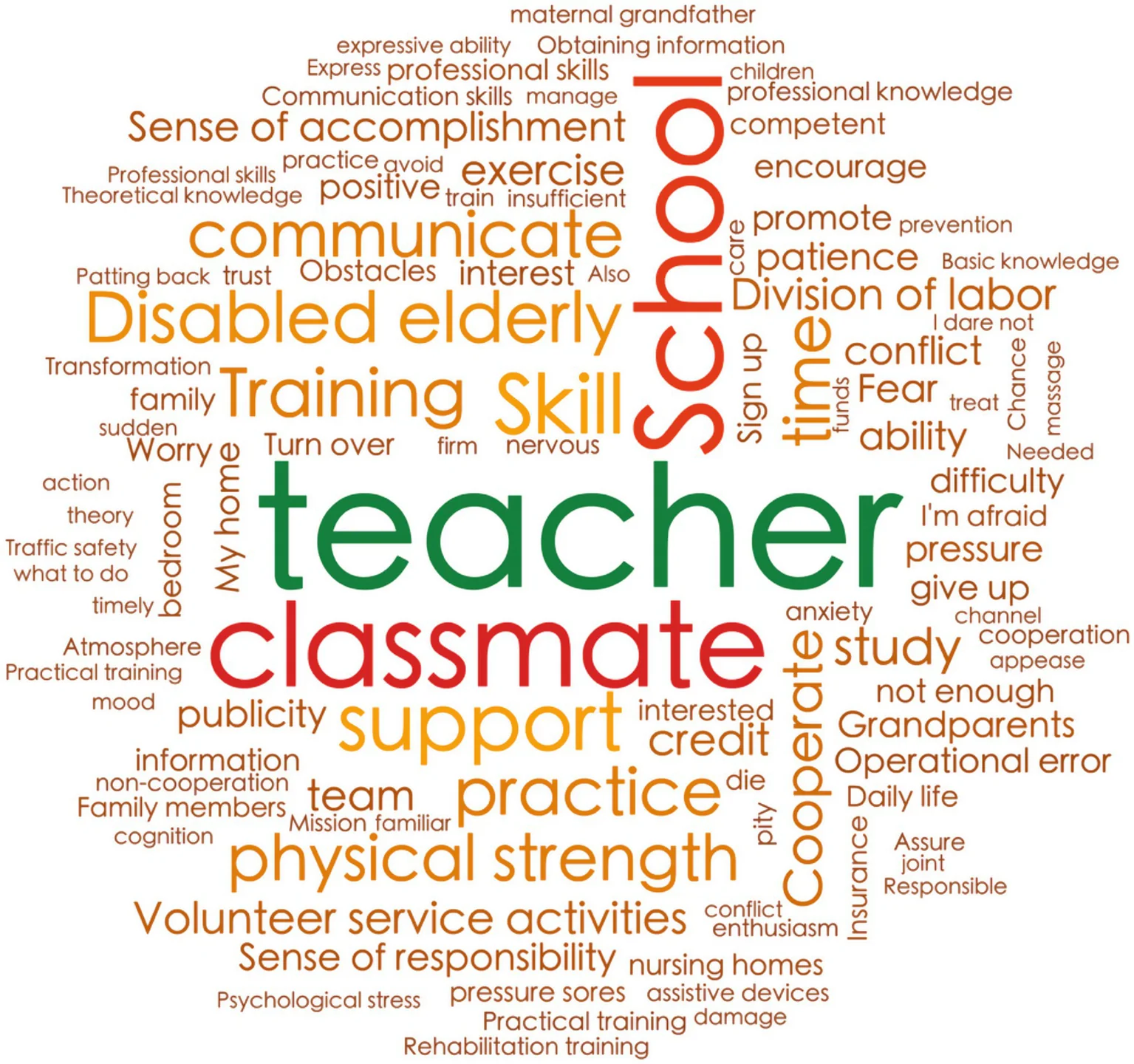
Word cloud.

**Figure 3:**
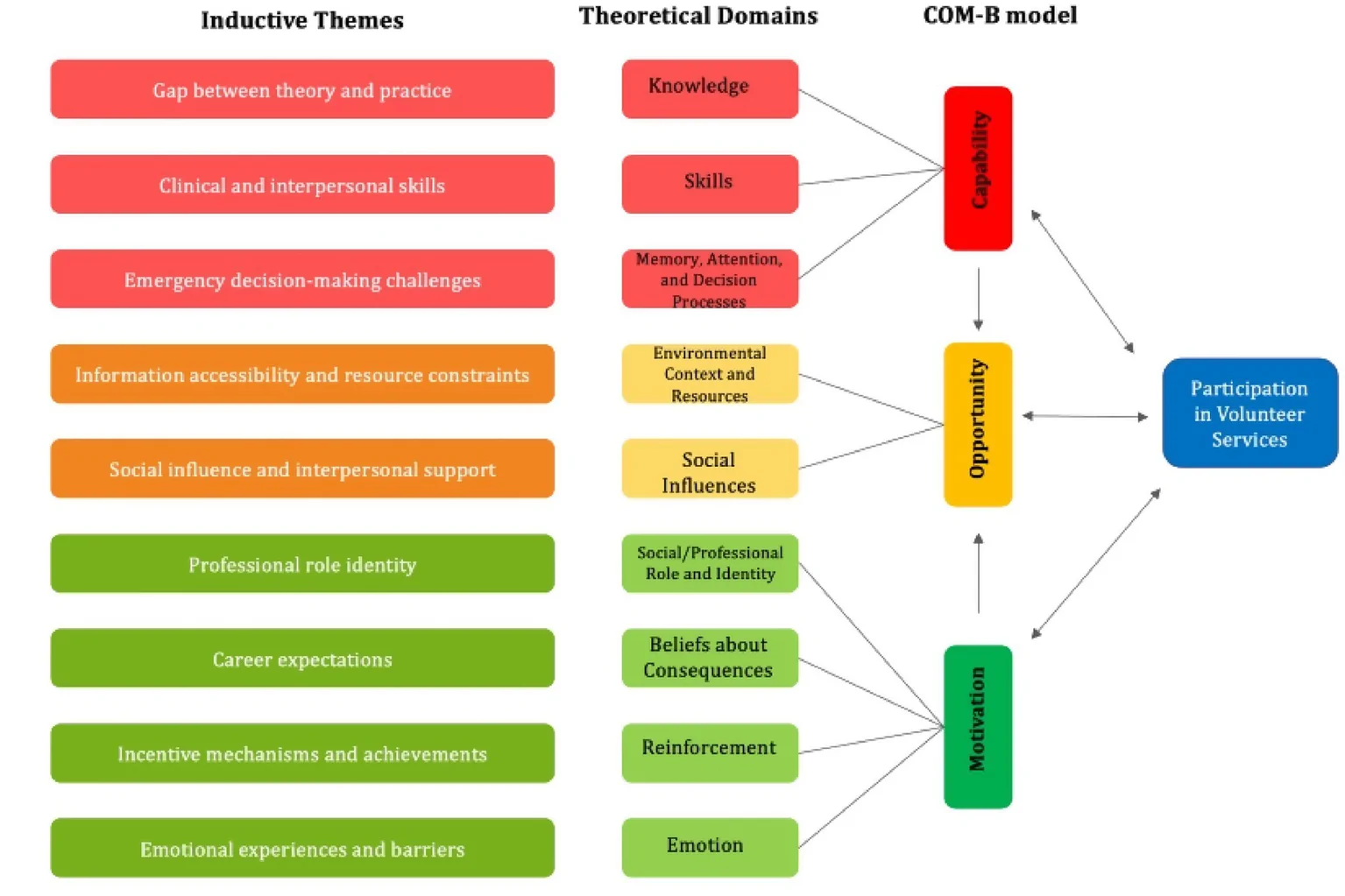
Influences on medical students’ participation in volunteer services for older adults with disabilities and their mapping onto TDF domains and COM-B constructs.

**Table 2:** Overview of analysis showing themes and sub-themes mapped to COM-B and TDF.

COM-B	TDF Domain	Themes	Sub-themes (barriers/facilitators)
Capability	Knowledge	1. Gap between theory and practice	Barrier: Insufficient specialized knowledge for geriatric care
	Skills	2. Clinical and interpersonal skills	Barrier: Lack of physical stamina for transfers Barrier/Facilitator: Challenges in professional communication
	Memory, Attention, and Decision Processes	3. Emergency decision-making challenges	Barrier: Anxiety/insufficient judgment during sudden health emergencies (e.g., falls)
Opportunity	Environmental Context and Resources	4. Information accessibility and resource constraints	Barrier: Fragmented recruitment information Barrier: Lack of funding for materials Barrier: Insufficient public attention and media coverage Facilitator: School-provided insurance and equipment
	Social Influences	5. Social influence and interpersonal support	Facilitator: Support from peers, teachers, and family Barrier: Resistance from older adults or a lack of cooperation from community staff
Motivation	Social/Professional Role and Identity	6. Professional role identity	Facilitator: Viewing service as a rehearsal for future professional responsibility
	Beliefs about Consequences	7. Career expectations	Facilitator: Belief that volunteering enhances resumes for future internships/jobs
	Reinforcement	8. Incentive mechanisms and achievements	Facilitator: “Second Classroom” credits and volunteer hour points Facilitator: Sense of achievement and being needed
	Emotion	9. Emotional experiences and barriers	Facilitator: Empathy transfer from personal elders Barrier: Negative emotions (fear of dirty tasks/death, anxiety)

#### 3.2.1 Capability

In the capability dimension, participants mostly discussed factors related to knowledge, skills, memory, attention, and decision processes.

- Gap between theory and practice
  Barrier: insufficient specialized knowledge for geriatric care

Most participants expressed that although they had learned basic theories in school, they still felt their professional knowledge and skill reserves were insufficient when facing the complex needs of older adults with disabilities, and this was consistently regarded as a major barrier. Confidence was particularly low in areas such as home suitability modifications, the use of complex rehabilitation devices, and responding to emergencies.

*Although I practiced turning over and patting the back in school, I still hesitated to do it when facing a real older adult. I worried that improper handling would cause them pain or discomfort*. (P7)

- Clinical and interpersonal skills
  Barrier: lack of physical stamina for transfers

Beyond knowledge, the physical demand of caring for older adults with disabilities (e.g., transferring patients from bed to wheelchair) was recognized as a barrier for individual participants, requiring team-based physical effort.

*Moving a patient who has completely lost their mobility… is very difficult for me alone; it requires teamwork*. (P3)

Barrier/facilitator: challenges in professional communication

Communication skills were regarded as a crucial capability, but also a significant challenge. Participants indicated that older adults with disabilities often have hearing difficulties, aphasia, or cognitive decline, which complicates communication. Participants struggled to accurately judge older adults’ needs, which impacted service decisions. On the other hand, these difficulties were gradually overcome through repeated contact, as participants grew more confident and familiar with the older adults they served.

*Some older adults do not express themselves clearly and cannot articulate their needs… We need to judge by observing the daily habits and actions of family members or the older adults*. (P5)

*Many older adults have speech impairments. When I do joint mobility training for them, I do not know if their resistance is due to pain or muscle spasms*. (P11)

*When I first started interacting with the older adults, I felt very flustered and at a loss. However, as I participated in more activities, I gradually got to know them, and communication became very easy*. (P4)

- Emergency decision-making challenges
  Barrier: anxiety and insufficient judgment during sudden health emergencies

Participants expressed anxiety about making rapid, accurate decisions during sudden health events outside a hospital setting, where monitoring equipment and real-time supervision were absent. In particular, they found it difficult to distinguish a fluctuation in a chronic condition from an acute attack.

*In a home environment, without monitoring equipment or real-time guidance from a teacher, I found it difficult to determine whether a sudden condition in an older adult was due to a fluctuation in a chronic condition or an acute attack, which terrified me*. (P6)

*I also need to be able to assess and handle emergencies*. (P8)

#### 3.2.2 Opportunity

The opportunity dimension covers environmental context, resources, and social influences.

- Information accessibility and resource constraints
  Barrier: fragmented recruitment information

The blockage of information channels posed a significant barrier to accessing necessary resources. There is a lack of centralized, open platforms for specific volunteer opportunities for older adults with disabilities. Many participants stated that it was difficult to obtain information about volunteer services, often relying on interpersonal recommendations rather than active public recruitment channels.

*Obtaining recruitment information is extremely difficult and fragmented, relying primarily on word-of-mouth from senior students. From a public health perspective, the lack of a standardized information dissemination platform directly leads to a mismatch between supply and demand, leaving many interested individuals unable to find a way to participate*. (P12)

Barrier: lack of sustained funding for materials

Beyond one-off support, a stable funding line covering daily necessities, nutritional supplies, and home modifications was absent, limiting the continuity and scale of services.

*For volunteers, funding is very important… For example, we want to renovate the housing of older adults with disabilities, but we lack funds*. (P2)

*I believe there is insufficient funding for material support, such as nutritional supplements and daily necessities for the older adults, as well as for purchasing supplies for activities*. (P9)

Barrier: insufficient public attention and media coverage

Participants also perceived weak public recognition of such services.

*Furthermore, there is a lack of social attention and media coverage*. (P13)

Facilitator: school-provided insurance and equipment

At the same time, the school lowered the practical risks of participation by providing safety protection, insurance, and equipment.

*Safety protection was very thorough. On campus, safeguards were well in place, and for off-campus activities, we were usually covered by insurance*. (P8)

- Social influence and interpersonal support
  Facilitator: support from peers, teachers, and family

Social influences, including support from peers, teachers, and family, played a positive role in promoting participation. The team’s collaborative atmosphere and the “leaders” demonstration effect could significantly enhance participants’ excitement.

*At first, my roommates were not interested… But after persisting for a while, I found I could influence them, and they often came to ask how they could join*. (P1)

Barrier: resistance from older adults and limited cooperation from community staff

Cooperation from community staff and the trust of the older adults themselves were inconsistent. Several participants reported facing resistance from older adults or a lack of cooperation from community workers during the initial integration into the community.

*During my volunteer work, I found that the level of cooperation from community staff directly determines work efficiency*. (P10)

*Initially, the older adults were resistant to us… but after serving them many times, I gained a sense of accomplishment, and they trusted me more*. (P5)

#### 3.2.3 Motivation

The motivation dimension highlighted the interplay between professional aspirations, emotional regulation, and external rewards.

- Professional role identity
  Facilitator: viewing service as a rehearsal for future professional responsibility

Regarding reflective motivation, participants generally demonstrated a strong sense of professional identity. As medical students, they regarded service as a way to consolidate their professional abilities and rehearse the responsibilities of their future roles.

*My major is clinical medicine… I want to improve some of my skills* and s*ense of responsibility through volunteering*. (P2)

- Career expectations
  Facilitator: belief that volunteering enhances resumes for future internships and jobs

Participants also anticipated instrumental career benefits, believing that such experience strengthened their competitiveness for later internships and employment.

*It helps my resume… and allows me to understand the general health industry earlier*. (P10)

- Incentive mechanisms and achievements
  Facilitator: “second classroom” credits and volunteer hour points

In addition to pure charity, external accreditation, notably the “Second Classroom” credit system and the accumulation of volunteer hour points, served as a concrete reinforcement for continued participation.

*There is a credit certification… it also adds points to volunteer hours*. (P1)

Facilitator: sense of achievement and being needed

Regarding automatic motivation, the sense of achievement and feeling of being needed were crucial reinforcement factors maintaining volunteering behavior.

*Seeing the older adults say ‘thank you’ countless times gave me a great sense of accomplishment, which also reflects the value of volunteer service*. (P16)

- Emotional experiences and barriers
  Facilitator: empathy transfer from personal elders

Furthermore, empathy served as a significant motivator, as numerous participants transferred their emotions for their own elders onto the service recipients.

*Seeing them is like seeing my own grandparents; I feel happy to give back, and it gives me a great sense of accomplishment*. (P4)

*I was raised by my grandfather when I was young… When they get old, we should have a responsibility to take care of them*. (P15)

Barrier: negative emotions such as fear of dirty tasks, fear of death, and anxiety

At the same time, distressing emotions such as fear (fear of dirty tasks, fear of fatigue, fear of facing death) and anxiety (worry about inability to solve problems) could trigger emotions that participants perceived as potential reasons to disengage.

*When I encounter the older adults feces, urine, or vomit, it might make me want to give up. Also, I’m afraid of facing death*. (P14)

## 4 Discussion

Based on the COM-B model and the TDF framework, this study identified and examined the facilitators and barriers to medical students’ engagement in volunteer services for older adults with disabilities. The findings indicate a complex mechanism influencing student engagement: while participants demonstrated significant motivation, primarily driven by their unique professional role identity, empathy for aging family members, and external incentives from the university, barriers within the Capability and Opportunity dimensions were particularly pronounced at the execution level. Specific obstacles included the gap between theoretical knowledge and clinical practice, communication deficits when interacting with older adults with disabilities, passive information accessibility, and substantial resource constraints regarding sustainable material support. This study suggests that while social support and institutional assurances facilitate participation to a certain extent, anxiety regarding professional skills and insufficient resources at the implementation level are the main obstacles hindering the sustainability of the service, presenting a practical dilemma of “high intention, low efficacy”.

The ability of medical students to translate theoretical knowledge into practical care for older adults with disabilities is crucial for effective volunteering (27). A primary barrier identified in this study was the significant gap between explicitly taught theoretical knowledge and the realities of clinical practice. Drawing on Polanyi’s (7) epistemological framework, what participants primarily lacked was tacit knowledge, namely the experiential knowledge required to navigate complex and dynamic care situations. Although participants possessed foundational medical theory, they expressed a significant lack of confidence in performing specialized care interventions, including the use of rehabilitation devices and managing home-based emergencies. For example, distinguishing muscle spasms from pain-induced resistance during physical therapy, as described by Participant P11, relies heavily on tacit knowledge acquired through sustained physical contact and observation, rather than on explicit textbook instructions. This corresponds with previous studies suggesting that students frequently encounter “transition shock” or anxiety when applying theoretical knowledge from the classroom to clinical practice (28). In contrast to the hospital environments described in previous studies, where professionals rely on clinical experience and immediate supervision (29), student volunteers in this study operated in community settings with limited professional oversight. This context explains the reported “fear of causing harm” and the hesitation to perform physical transfers. Consequently, there is a highlighted need for simulation-based training specifically designed for community and home-care scenarios, rather than training focused purely on acute hospital care (30). Furthermore, communication impairments in older adults with aphasia or cognitive decline were recognized as a significant obstacle to capability. This suggests that current medical curricula may emphasize clinical pathology at the expense of the essential communication skills needed for interacting with vulnerable populations, resulting in a competency gap that hinders student engagement (31).

Environmental factors and resource limitations emerged as significant obstacles, eclipsing the support offered by the university. While the university provides institutional support such as insurance and equipment, the opacity of recruitment channels created a high entry barrier. The findings indicate that participants depend on passive information gathering through personal networks, mirroring how healthcare professionals in previous studies on incidental learning instead of formal frameworks (32). This information imbalance creates a barrier, excluding students who have strong desires but lack interpersonal networks. Additionally, the lack of sustained funding for material support, such as home modifications and daily necessities, limits the scope of practice. This finding aligns with extensive studies on community volunteering, demonstrating that a lack of tangible resources significantly reduces volunteers’ perceived impact and self-efficacy (33). This paucity of resources forces participants to merely cope with existing barriers rather than resolving them, fostering a sense of professional powerlessness that ultimately undermines the sustainability of their service. Therefore, future volunteer programs must prioritize the allocation of project-specific funding to transform basic companionship into substantial assistance, thereby restoring students’ sense of professional efficacy.

The findings regarding motivation reveal a complicated relationship between career ambition and emotional reality. On the one hand, professional role identity served as a robust facilitator for participants perceiving volunteering not solely as a charitable act, but as essential preparation for their future medical careers. This is consistent with studies suggesting that associating volunteering with professional development promotes sustained engagement (34). Furthermore, empathy transfer served as a powerful intrinsic motivator, where participants treated older adults with the same care intended for their grandparents (35). Nevertheless, these facilitators encounter substantial psychological obstacles. This study revealed the intense hesitation and resistance among participants to assist older adults with hygiene and environmental cleaning tasks, including bathing, body sponging, and incontinence care, alongside anxiety related to hospice care and death. In contrast to professional healthcare workers who may be desensitized or trained to handle mortality (36, 37), student volunteers frequently lack the psychological resilience to process these stressors. This emotional burden functions as a barrier similar to the “lack of accountability” mentioned in prior research (38), where psychological pressure leads to avoidance behaviors. This highlights the pressing necessity for psychological support systems, such as debriefing sessions, to assist students in processing their emotional experiences and prevent compassion fatigue.

## 5 Limitations

There were several limitations in this study. First, more than 50% of the participants were female, which limited the gender diversity in this study to some extent. However, this distribution largely reflects the demographic reality of nursing and medical schools in China, and the researchers did their best to recruit male participants to adhere to the principle of maximum variety in the data. Moreover, the participants were recruited from two medical universities in Southwest China, which may limit the generalizability of the findings to other regions with different economic or cultural contexts. Nevertheless, the researchers employed the theoretical frameworks of the COM-B model and TDF to ensure the theoretical rigor and transferability of the observed barriers and facilitators.

## 6 Conclusion

These findings provide valuable insights into the complex barriers and facilitators that impact medical students’ sustained participation in volunteer services for older adults with disabilities, providing crucial insights into an area of research within medical education and community care. The analysis contributes to the broader body of knowledge on volunteer management and emphasizes the need for a comprehensive support system that includes simulation-based skills training, psychological resilience development, and strategic resource allocation to bridge the gap between students’ strong motivation and their practical implementation. Conceptualizing the findings through the COM-B model and the TDF framework clarifies the conflict between students’ high professional ambitions and the existing structural and capability shortcomings. This theoretical perspective demonstrates that intrinsic motivation, however robust, is insufficient to counterbalance the lack of specialized skills and operational resources. The importance of this study resides in its capacity to guide focused strategies and policy modifications that can transform volunteer programs from passive companionship into skilled, professional assistance. Ultimately, overcoming these obstacles will enhance the professional competence of future healthcare providers and substantially improve the quality of care and life for older adults with disabilities.

### 6.1 Limitations and future directions

This study was limited by its regional sample focus and exclusive perspective of medical student volunteers. Future research should adopt multi-centre designs, include community staff and older adults with disabilities, and test COM-B-informed interventions to translate findings into practice.

## Data Availability

The raw data supporting the conclusions of this article will be made available by the authors, without undue reservation.
