# Supplementary material for: Perceived barriers and facilitators to medical students participating in volunteer services for older adults with disabilities: a qualitative descriptive study
Source: Front Public Health. 2026 Oct 13;14:1918396. doi: 10.3389/fpubh.2026.1918396 (PMC13710319; doi:10.3389/fpubh.2026.1918396)
Supplement: Supplementary file 2 [file Table_1.docx]

**Table S1.** Interview outline.

| **COM-B Framework** | **Guiding Questions** |
| --- | --- |
| **Capability** | 1. What professional knowledge and skills do you think are necessary to effectively serve disabled elderly people? |
|  | 2. Do you possess this knowledge and these skills? What are the sources of these skills? |
|  | 3. What difficulties do you encounter when serving and communicating with disabled elderly people? How do you cope with them? |
|  | 4. How is the collaboration with the service team members? Are there any problems, such as unclear division of labor or poor coordination? |
|  | 5. How do you assess the physical requirements of this type of volunteer service? Do you believe your physical strength is sufficient? |
| **Opportunity** | 6. Do you know through which channels you can register for this type of volunteer service? How easy is it to obtain this information? |
|  | 7. Do you think there is a conflict between your personal studies, schedule, and volunteer service? Do you think the necessary transportation, safety protection, and other guarantees for participating in the service are adequate? |
|  | 8. What is the atmosphere like among your classmates who participate in this type of volunteer service? How will their participation (or non-participation) affect your choice? |
|  | 9. To what extent do you think factors such as your school/college/teachers/society encourage or support your participation in these activities? What is your family’s attitude? |
| **Motivation** | 10. What was your initial motivation for participating in volunteer services for disabled elderly? Was it out of interest, a sense of responsibility, or other reasons? |
|  | 11. What specific benefits do you see for your personal or professional development from participating in volunteer services for disabled elderly? |
|  | 12. What are your feelings or emotions when serving disabled elderly? Do you feel pressure, anxiety, or a sense of accomplishment during the service process? |
|  | 13. During the participation process, what situations might make you want to give up? What are the attitudes of the elderly you serve and the community? |
| **Summary** | What do you think is the biggest factor currently hindering people from participating in volunteer services for disabled elderly? |
|  | What conditions or support do you think are most needed to promote more active participation? |
